# Updates in the Clinical Development of Epacadostat and Other Indoleamine 2,3-Dioxygenase 1 Inhibitors (IDO1) for Human Cancers

**DOI:** 10.3389/fonc.2018.00423

**Published:** 2018-10-04

**Authors:** Takefumi Komiya, Chao H. Huang

**Affiliations:** ^1^Parkview Cancer Institute, Fort Wayne, IN, United States; ^2^Division of Medical Oncology, University of Kansas Medical Center, Fairway, KS, United States; ^3^Subspecialty Medicine, Division of Hematology & Medical Oncology, Kansas City VA Medical Center, Kansas City, MO, United States

**Keywords:** immuno-oncology, IDO (indoleamine 2,3-dioxygenase), PD-1, tryptophan, clinical trials as topic, IDO1 inhibitor

## Abstract

Recent application of immunotherapy in clinical oncology revolutionized our management of advanced human cancers. Check point inhibitors targeting CTLA4 and PD-1/PD-L1 axis are immunotherapeutic agents currently available to treat a variety of cancers. However, a novel therapeutic approach is needed to further improve patient outcome with these agents. Indoleamine 2,3-dioxygenase 1 (IDO1) is a rate-limiting enzyme in the metabolism of essential amino acid tryptophan in the peripheral tissue. IDO1 is overexpressed in human cancer cells and suppresses effector T cell function and promotes regulatory T cells (Tregs). Overexpression of IDO1 is associated with poor patient survival in several types of human cancer. These findings indicate that IDO1 is a promising target that can improve the treatment outcome in the field of Immuno-oncology. Several orally available IDO1 inhibitors including Epacadostat have entered human clinical trials over the last few years without a major safety concern. Although there is no objective response in single-agent trials, combination regimens with PD-1 inhibitors appear to exceed the activity of PD-1 inhibitors alone. Recent phase III ECHO 301 trial testing the combination of Epacadostat with Pembrolizumab in melanoma did not show superior outcome compared to Pembrolizumab alone. This lead to halting of other phase III trials using IDO1 inhibitors. In this minireview, we will discuss the recent clinical development of Epacadostat and other IDO1 inhibitors.

## Introduction

Cancer remains one of the leading causes of mortality worldwide, affecting 8.8 million people annually (1). Immune checkpoint inhibitors (CPI) enhance the T-cell mediated anti-tumor effect by disrupting the interaction between the PD-1 receptor and PD-L1 ligand. They were recently approved for a wide variety of cancer types and changed the landscape of therapy in cancer (2). These include nivolumab (N)/pembrolizumab (P) (anti-PD-1) and atezolizumab/durvalumab (anti-PD-L1) (3–6). Ipilimumab is the only clinically available anti-CTLA4 inhibitor with FDA approval limited to melanoma (7). As more immune targets are identified, many novel immune therapies to enhance T-cell function entered clinical trials.

**Figure 1:**
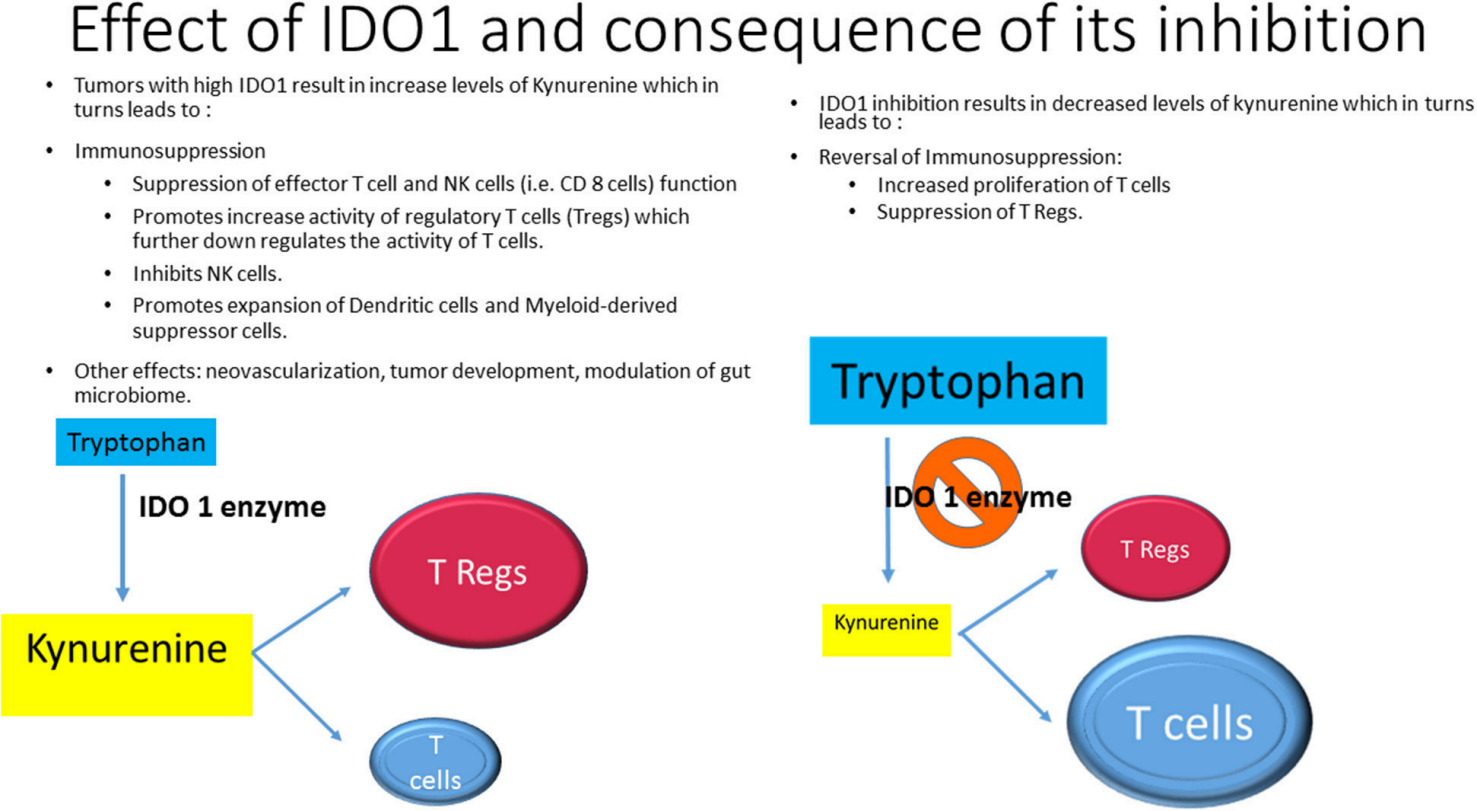
Effects of IDO1 and consequence of its inhibition.

Indoleamine 2,3-dioxygenase1 (IDO1), IDO2, and are rate-limiting enzymes in the metabolism of essential amino acid tryptophan (Try), converting it to kynurenine (Kyn), in the peripheral tissue. Tryptophan 2,3-dioxynase (TDO) is in hepatic tissue. The increased activity of IDO1 leads to suppression of effector T cell (i.e., CD 8 cells) function and increase the activity of regulatory T cells (Tregs) which further downregulates the activity of T cells. In addition, IDO1 inhibits NK cell function and promotes the expansion and activation of dendritic cells (DC) and myeloid-derived suppressor cells (MDSC). Other effects include neovascularization, tumor development and modulation of gut microbiome (8) (Figure 1). Several IDO1 inhibitors are currently under preclinical or clinical investigation (9). This minireview will primarily focus on recent clinical research of Epacadostat (E) and other IDO1 inhibitors and discuss available data and future developments.

## The role of IDO1 in the regulation of cancer immunity and preclinical development of IDO1 inhibitors

Oncogenesis is regulated by the interaction between cancer and various immune cells (9). Effector T cells such as CD8+ cells play a dominant role in negatively controlling cancer development, whereas T regulatory cells (Tregs) suppress immunogenic response and allow immune escape of cancer cells. Increased IDO1 activity leads to accumulation of Kyn which suppresses NK cell/effector T cell function and activates Tregs (9). Its overexpression has been observed in many types of human cancers and it is associated with poor survival (10–13). IDO1 has been described as a novel therapeutic immune target in recent years (14).

Researchers at NewLink Genetics developed a tryptophan mimetic 1-methyl-tryptophan (1MT) which exists in two stereoisomers with different biological properties (15). The L isomer (1-L-MT) is the more potent inhibitor of IDO1, whereas the D isomer (1-D-MT, Indoximod) exhibited significantly higher anti-tumor activity by reversing T-cell function and synergizing with chemotherapy agents (16) but is a more selective IDO2 inhibitor (17) which metabolizes tryptophan much less efficiently than IDO1 (18, 19). Recent studies showed that Idoximod is an indirect inhibitor of IDO1 pathway by reversing mTORC1 inhibition induced by tryptophan depletion (20, 21).

Epacadostat (INCB024360) is an orally available reversible competitive potent IDO1 inhibitor and has entered clinical studies (22). It showed a potent anti-IDO1 activity with *in vitro* enzymatic and cell-based assay with an average IC50 value of 71.8 and 10 nM, respectively. It has a >1,000-fold selectivity for IDO1 compared to IDO2 and TDO. E increased T/NK cell proliferation, expanded CD86 high DCs, increased IFN-gamma production and suppressed Tregs *in vitro*. *In vivo*, E also suppressed tumor growth in immunocompetent but not in immunodeficient mice, although plasma Kyn/Try levels were significantly suppressed in both models.

Other IDO1 inhibitors in clinical development include Navoximod (NLG-919) which is an oral IDO1 and TDO inhibitor; BMS-986205 which is an irreversible IDO1 inhibitor; PF-06840003 and BGS-5777 which are IDO1 inhibitors that can cross blood-brain barrier (8). IOM2983, RG70099, NLG802, HTI-1090, KHK2455 are still in early development (21, 23–26).

## Results in clinical investigation

### Single-agent studies

A phase I study in 48 patients with advanced solid tumor tested oral Indoximod at a dose ranging from 200 mg once daily to 2,000 mg twice daily (16). Idoximod was well tolerated and no patients discontinued treatment due to toxicity. Maximum tolerated dose (MTD) was not reached. Of note, three patients in the lowest dose level developed grade (G) 2 hypophysitis were previously treated with CPI (2 with Ipilimumab, 1 with anti-CD40 inhibitor). Efficacy analysis demonstrated five patients with stable disease (SD) of >6 months duration.

A dose escalation phase I study in 52 patients with advanced solid tumors tested E at a dose ranging from 50 mg once daily to 700 mg twice daily (BID) (27). E was well tolerated and MTD was not reached. The most common adverse events (AEs) was fatigue and others are described in Table 1. Seven patients discontinued treatment due to AEs, including 2 patients with dose-limiting toxicities (DLTs). PK studies showed a half-life of 2.4–3.9 h with modest variability between subjects and non-significant effect with ingestion of food with high-fat content. PD study demonstrated decreased in plasma Kyn level and Kyn\ Try ratios at all doses. The best response was SD in 18 of 52 patients lasting 16 weeks or longer seen in 7 patients (13.5%). As single drugs, these 2 agents were only able to produce SD with tolerable side effects.

**Table 1:** Selected trials targeting IDO1.

**Agent**	**Combination**	**Results of clinical studies**		**Safety**
Idoximod	Single Agent NCT00567931	Phase I ORR 10% (5/48)		Fatigue (56%), Anemia and Anorexia (37.5%), Dyspnea (35.4%) Cough (33.3%), Nausea (29%)
	Docetaxel (NCT01191216)	Phase I ORR 18% (4/22)		Fatigue (58%.6), Anemia (51.7%), Hyperglycemia (48.3%), Infection (44.8%), Nausea (41.4%)
	Nab-Paclitaxel/Gemcitabine (NCT0207781)	Phase I/II ORR 37% (11/3)		Colitis (3%)
	Pembrolizumab (NLG2103) NCT02073123	1^st^ line Melanoma ORR of 55.7% (39/70, 36 confirmed). mPFS was 12.4 m		Fatigue (60%), headache (33%), nausea (32%), arthralgia (28%), diarrhea (28%), pruritus (26%), rash (23%), and cough (21%), whereas G3 AEs: fatigue, diarrhea, and rash (all 1 case each)
	Adenovirus transduced dendritic cell vaccine. (NCT01042535)	Breast Ca ORR 38% (1CR, 7 PR / 21) and SD 9% (2/21). The mPFS was 6.8 weeks. Early termination		G1, G2: fatigue, anemia, transient lymphopenia, nausea, anorexia
	Temozolomide (NCT02502708)	NR	
	Idarubicin/Cytarabine (NCT02835729)	NR	
	Temozolomide/bevacizumab (NCT02052648)	NR	
	Sipuleucel-T (NCT01560923)	NR	
	Ipilumumab/Nivolumab/Pembrolizumab (NCT03301636)	NR		
Epacadostat	Single Agent (NCT01195311)	SD in 18 of 52		Fatigue (69.2%), nausea (65.4%), loss of appetite (53.8%), and vomiting (42.3%) 1 DLT at of 300 mg BID (G3, radiation pneumonitis); 1 DLT at 400 mg BID (G 3, fatigue)
	Pembrolizumab (ECHO 202) NCT02178722	1^st^ Line Melanoma ORR 58% (11/19)	Pembrolizumab	
			33%
		2^nd^ line NSCLC ORR 35% (14/40)	18%	15% had grade ≥3 TRAEs lipase (asymptomatic), rash (3% each) and 3% of patients discontinued treatment due to TRAEs
		2^nd^ Line H&N ORR 34%(13/38)	16–18%	
		Urothelial Ca ORR 35% (14/40)		
		Renal Cell Ca ORR 33% (10/30)		
	Nivolumab (ECHO 204) NCT02327078	Melanoma 63% (25/40) 2^nd^ Line H&N 23% (7/31)	Nivolumab* 40%13%	Grade ≥3 TRAE in E 100 mg and E 300 mg subgroups (Rash 10% and 12%)
	Pembrolizumab and chemotherapy (ECHO 207) NCT03085914 Pembrolizumab+E vs. Pembrolizumab (ECHO 301 Melanoma) NCT02752074 Pembrolizumab/chemotherapy (NCT 02862457) Azacytidine/Pembrolizumab NCT02959437 Pembrolizumab/chemotherapy ECHO 306 NCT003322566 Nivolumab/chemotherapy (NCT02327078)	NR Phase II various cancers Halted—Negative Trial NR—solid tumors NR—solid tumors NR—Lung cancer NR		
Navoximod	Single Agent (NCT02048709) Atezolizumab (NCT 02471846)	NR NR		
PFS-06840003	Single Agent (NCT02764151)	NR in Malignant Gliomas		
BMS 986205	Nivolumab (NCT03329846)	NR in Melanoma, advance ca		
	Nivolumab/Ipilimumab (NCT 02658890)	ORR 32% (Bladder), 14% (Cervical) PD-L1>1% ORR 46% (Bladder), 25%(Cervical)		Fatigue, Nausea (18%), Anorexia (13.6%) Vomiting (6.8%)
	Nivolumab/Cetuximab/Chemotherapy Nivolumab/chemotherapy	Halted H&N ca Halted Lung		

NR, not reported; SD, stable disease; G3, grade 3; DLT, dose limiting toxicity; TRAE, treatment related adverse event. *Historical data for comparison

### Combination studies with indoximod (NLG-8189)

NLG2103 was a phase II study of Indoximod in combination with CPI in 94 patients with advanced melanoma (28). P was given at 2 mg/kg IV every 3 weeks with oral Indoximod at 1,200 mg BID in a 21-day cycle until intolerance or disease progression. Fatigue was the most common AE and other are listed in Table 1. ORR was 55.7% and median progression-free survival (PFS) was 12.4 m (29). A phase III trial NLG 2107 using Indoximod or Placebo plus P or N in patients with unresectable or metastatic melanoma is ongoing (30). Several studies are combining Idoximod with other systemic therapy such as cancer vaccines or cytotoxic chemotherapeutic agents. Based on the pre-clinical mouse model of breast cancer demonstrating the synergistic effect of Indoximod in combination with Paclitaxel on tumor regression (31), a phase I study of Indoximod in combination with Docetaxel was conducted in 27 patients with advanced solid tumors with ORR of 18%. The combination was well tolerated with no increased toxicity relative to single-agent docetaxel. Other ongoing studies combined Idoximod with gemcitabine/nab-paclitaxel, temozolomide, sipuleucel-T, or adenovirus-p53 transduced DC vaccine (32–35). The last study in breast cancer patients (NCT01042535) was terminated early and the data is not evaluable, making it difficult to assert the utility of this approach.

### Combination studies with epacadostat

IDO1 inhibitors could enhance the effect of CPI by preventing immune escape through suppression of Kyn level and consequent suppression of Tregs and activation of T cells (Figure 1). Several studies were initiated to prove this concept.

ECHO202 was a multi-disease cohort, phase I/II study to define MTD and efficacy/safety in advanced solid tumor. Patients were treated with P at 2 mg/kg or 200 mg every 3 weeks in combination with oral E 25–300 mg BID until disease progression or intolerance. The results of the melanoma cohort showed an ORR of 58% (36). The efficacy data of other cohorts and AEs are described in Table 1. The response in different types of cancers, ranged from 33 to 58% and they were higher than historical single agent P studies (16 to 33%) (Table 1) (37–40). Safety analysis in 244 patients in the ECHO 202 study is also detailed in Table 1. Most were asymptomatic lipase elevation and there were no treatment-related deaths (41). Based on overall safety and efficacy, E 100 mg BID was selected as the recommended phase II dose.

ECHO204 was another multi-disease cohort, phase I/II study of E in combination with N with similar design and outcome of ECHO202. Preliminary results reported in 2017 also indicated that the combination has minimally increased toxicity and promising efficacy data compared to studies using single agent N (Table 1) (42). Overall, these early phase studies suggest that despite the lack of single agent anti-tumor activity, the addition of E to CPI are well-tolerated and produced relatively higher ORRs compared to studies using single agent CPI. The encouraging results of these 2 studies lead Incyte to launch several phases III registration trials to define the efficacy of E in combination with P and/or chemotherapy in solid tumors (ECHO 301–310) (43–51). Several of them are also adding E and CPI to chemotherapy (Table 1). Other investigator-initiated/sponsored trials using various combination partner drugs are also underway, including Sirolimus, Azacytidine, cancer vaccine, Itacinitib (52–55).

Recently, the enthusiasm with IDO1 inhibitors was dampened with the negative results of ECHO-301, a phase III trial in patients with unresectable or metastatic melanoma comparing E with P vs. P alone. The study did not meet its primary objective of improvement in PFS (56). The reasons for this negative trial are undefined. Further research regarding possible imbalance of patient groups, including pre-treatment use of prednisone which could decrease the response of P (57), differences in tumor mutation burden and patient's microbiota (58) could help to elucidate the reason for this negative trial. Other possibilities to explore are compliance with oral administration of E, level of inhibition of Kyn/Try ratio and development of inflammation as mechanism of resistance (59).

### Other IDO1 inhibitors in clinical development

BMS-986205, a novel, and more potent IDO1 selective inhibitor, is currently in development by Bristol-Myers Squibb. A phase I/II study of BMS-986205 alone or in combination with N was reported in 2017 (60). Patients with advanced cancers were treated at 25–200 mg once daily for a 2-week lead-in period, followed by the addition of N 240 mg IV every 2 weeks. Among the 42 evaluable patients, all treatment-related adverse events were G1-2 except three G3 toxicities. Serum Kyn was substantially reduced at all doses with >60% mean reduction at the 100 and 200 mg doses. Although efficacy result has not been reported, BMS-986205 showed a significant reduction in intratumoral Kyn in samples from 13 patients. Limited data of clinical efficacy were presented with a dramatic reduction in the size of the target lesion in 3 patients: head and neck cancer (63%), clear cell renal carcinoma (42%) and triple-negative breast cancer with 8 prior therapies (48%). A 3-arm phase I/II trial in combination with nivolumab at 2 doses or with Nivolumab plus Ipilimumab is underway.

Regrettably, 2 phase III trials of this agent in combination with Nivolumab/chemotherapy in H&N cancer and another in Lung cancer were halted after the press release of ECHO-301 negative results. Other new IDO1 inhibitors in clinical development do not have results to review (Table 1).

### Novel approaches to test IDO1 inhibitors in clinical trials

In addition to testing IDO1 inhibitors in advanced malignancies, researchers are investigating the role of IDO1 inhibitors in other clinical settings. A phase I trial combining E with external beam radiation therapy (XRT) and investigational TLR9 agonist SD-101 in patients with lymphoma (61) is based on the synergistic effect of XRT and CPI (62). In early-stage disease, a phase I/IIb trial is ongoing to assess the safety, toxicity, and PFS in patients who had undergone maximum debulking surgery for ovarian cancer using E (63) based on the improvement of recurrence-free survival using P/N as adjuvant therapy in resected melanoma (64, 65) and durvalumab in stage III lung cancer (6).

Similar to neoadjuvant or window-of-opportunity trials in early-stage disease (66, 67), IDO inhibitors are under evaluation in several diseases to primarily determine the pathologic effect in the tumor by collecting pre- and post-treatment tissue materials (68, 69). Future studies integrating peptide vaccine, anti-angiogenesis agents with IDO1 inhibitors are also of interest in view of its potential synergistic pathways (8).

## Conclusion

IDO1 inhibitors have been in active clinical investigation and preliminary results suggest that they are well tolerated and produce additive efficacy when combined with CPI despite low levels of activity as a single agent. Epacadostat, a highly selective IDO1 inhibitor had increased response when combined with N or P in phase I/II trials and entered phase III trials. However, the negative results of the phase III trial ECHO 301 raised the questions about the efficacy of this combination strategy. Given the potential of IDO1 inhibitors to enhance immunologic function, it would be premature to abandon its development. Further research in combination with other synergistic agents and molecular characterization of responders and non-responders will be vital in the future of this agent in the field of immunotherapy.

## Disclosure

The University of Kansas Research Institute has initiated a contract negotiation for an investigator-sponsored clinical trial sponsored by Incyte (51).

## Author contributions

TK and CH contributed conception and design of the study. TK wrote the first draft of the manuscript. All authors contributed to manuscript revision, read and approved the submitted version.

### Conflict of interest statement

CH has received research funding from Bristol Myers Squibb, Merck, Bayer, Genentech, Lilly, Novartis and BPI; served as co-investigator in a clinical trial sponsored by Incyte; and received consulting fee from Ipsen. The remaining author declares that the research was conducted in the absence of any commercial or financial relationships that could be construed as a potential conflict of interest. The reviewer KM and handling Editor declared their shared affiliation.
